# Long-term trends in the burden of pulmonary arterial hypertension in China and worldwide: new insights based on GBD 2021

**DOI:** 10.3389/fmed.2024.1502916

**Published:** 2025-01-07

**Authors:** Zhehao Xu, Jia Ding, Ruiyun Liang, Shuangfeng Xie

**Affiliations:** ^1^Department of General Medicine, Sun Yat-sen Memorial Hospital, Sun Yat-sen University, Guangzhou, China; ^2^Department of Hematology, Sun Yat-sen Memorial Hospital, Sun Yat-sen University, Guangzhou, China; ^3^Department of Respiratory Medicine, Sun Yat-sen Memorial Hospital, Sun Yat-sen University, Guangzhou, China

**Keywords:** age-period-cohort analysis, pulmonary arterial hypertension, mortality, prevalence, projection, global burden of disease

## Abstract

**Background:**

Pulmonary arterial hypertension (PAH) poses a significant health challenge globally, with China experiencing a notable increase in its burden. Understanding the trends and factors contributing to PAH is crucial for developing effective public health strategies.

**Methods:**

This study utilized data from the Global Burden of Disease (GBD) 2021 database to estimate the burden of PAH in China and worldwide from 1990 to 2021. A Bayesian age-period-cohort (BAPC) model was employed to analyze differences in PAH burden across age, gender, and time periods, and to project global epidemiological trends until 2036.

**Results:**

From 1990 to 2021, the incidence and prevalence of PAH in China increased by 80.59% and 86.74%, respectively. The age-standardized incidence rate (ASIR) and age-standardized prevalence rate (ASPR) showed an annual percentage change (AAPC) of -0.07% and 0.25%, respectively. Conversely, the age-standardized disability-adjusted life year (DALY) rate and age-standardized mortality rate (ASMR) have been declining since 1990, with AAPC of -1.90% and -1.26%, respectively. Females and the 50–70 years age group experienced a higher PAH burden compared to males. Projections indicate that ASPR, ASMR, and age-standardized death rate (ASDR) will stabilize with minimal variation over the next decade.

**Discussion:**

The findings highlight the age-related burden of PAH in China, particularly affecting older populations and women. The projected stabilization of PAH metrics over the next decade underscores the need for continued monitoring and targeted interventions. This study’s comprehensive analysis of PAH burden over three decades provides valuable insights for policymakers and healthcare providers, necessitating concerted efforts to address this critical health issue.

## Highlights

- This article is the first to report the epidemiology of PAH in China and worldwide.
- Females had a higher PAH burden than males, with older age groups most affected.
- PAH’s public health challenge in China is significant due to demographic trends.

## Introduction

Pulmonary arterial hypertension (PAH), a less common subtype of pulmonary hypertension, is distinguished by a progressive narrowing of the small pulmonary arteries (1, 2), typically results in right heart failure and can be fatal (3). In terms of hemodynamics, PAH is diagnosed when right heart catheterization reveals a mean pulmonary artery pressure above 20 mmHg (4, 5). The process of vascular remodeling (6) in the pulmonary arteries, characterized by the expansion of smooth muscle and endothelial cells, is acknowledged as a significant contributor to PAH pathogenesis (7), affecting the pulmonary arterial circulation which is characterized by flow, high surface area, and low resistance (8, 9). Common symptoms in PAH patients include labored breathing during exertion, fatigue, chest discomfort, and peripheral edema, with syncope occurring in severe instances, contributing to a delayed diagnosis in many patients. The detection of PAH is further complicated by the presence of associated conditions such as liver disease, connective tissue disorders, or a history of drug or toxin exposure, which should raise clinical suspicion for PAH (10, 11).

PAH represents a significant yet often overlooked global health concern, affecting individuals across all age ranges with a notable impact on the elderly (12). This condition frequently occurs alongside chronic respiratory diseases, such as chronic obstructive pulmonary disease and interstitial lung disease, with a notably elevated prevalence in patients with advanced stages of these illnesses (7). PAH impacts approximately 25 individuals per million in Western nations (13), with an annual incidence ranging from 2 to 5 cases per million people (14). The prevalence of other categories within the pulmonary hypertension spectrum varies by etiology and disease status, and it is likely that it is significantly underreported on a global scale (15). PAH complicates numerous common cardiopulmonary diseases, leading to increased morbidity and mortality rates. Individuals with PAH engage extensively with healthcare resources. The prevalence of PAH in China is on the rise, correlating with the nation’s economic growth and evolving lifestyle patterns (16). As the most populous country globally, the escalating burden of PAH has garnered considerable attention from the medical community in China (7, 16).

The latest Global Burden of Disease (GBD) 2021 findings have now replaced earlier GBD iterations (17), encompassing metrics such as prevalence, incidence, mortality and disability-adjusted life years (DALYs) (17–19). However, to our current awareness, no existing literature has specifically detailed the PAH burden stratified by age, gender, and temporal changes, nor projected the epidemiological patterns over the subsequent 15 years (20). We hypothesize a significant association between gender and PAH prevalence, reflecting potential disparities due to biological, environmental, or societal factors. We also anticipate variations in PAH burden across different age groups, with age-specific trends that may be influenced by comorbidities and physiological changes associated with aging. Furthermore, we expect to observe temporal shifts in PAH epidemiology, influenced by improvements in medical care, changes in lifestyle, and environmental factors. Finally, we predict that our epidemiological forecasts will offer valuable insights, aiding in the development of proactive public health strategies to address the anticipated rise in PAH incidence and mortality.

## Materials and methods

### Data source

This study’s data were sourced from GBD 2021, which documents the prevalence, incidence and mortality rates for 371 conditions and injuries, differentiated by age and gender (21). GBD compiles data from diverse aspects, such as longitudinal studies, clinical trials, population surveys, and additional research, to measure patterns and shifts in risk factor exposures, aiming to prevent disease-related health issues and early mortality (22). According to the etiology and hemodynamic characteristics of pulmonary hypertension, it was divided into five clinical subgroups by WHO, this study focused on PAH (group 1), formerly known as Primary PH. Other PH subgroups (group 2–5) were not included because of their different pathophysiology, treatment, and natural course of disease. In the 10th revision of the International Classification of Diseases (ICD-10), PAH is categorized under codes I27.001 to I27.003.

For the majority of health conditions and injuries, the prevalence and incidence rates were determined utilizing DisMod-MR 2.1, a meta-regression tool for disease modelling. Mortality associated with various diseases and injuries was estimated using the Cause of Death Ensemble model (CODEm). DALYs, a metric aggregating YLDs and YLLs, were derived by summing these components. YLDs were computed by applying the prevalence of each condition to its sequelae, while YLLs were determined by applying the mortality figures. Rates were standardized to the GBD’s global reference population.

The projections of the population were derived from the United Nations’ World Population Prospects as revised in 2019. Ethical review was waived by the relevant institutional committee. All graphical representations were generated using R software, version 4.2.3.

### Joinpoint regression analysis

We utilized the GBD data set to obtain data on the prevalence rate, incidence rate, mortality rate, DALYs and age-standardized prevalence rate (ASPR), age-standardized incidence rate (ASIR), age-standardized DALY rate (ASDR) and age-standardized rates of mortality (ASMR) for China and the world from 1990 to 2021. The average annual percentage change (AAPC) and its corresponding 95% confidence interval (95% CI) were determined using Joinpoint Software 4.9.1 from the National Cancer Institute, Rockville, MD, United States. The statistical significance was evaluated through the Monte Carlo simulation method, with a *p*-value threshold of <0.05.

### Age–period–cohort analysis and projection of model development

The age-period-cohort model, a widely utilized statistical method for revealing underlying patterns in disease occurrence and death rates (23), was applied in our analysis. We categorized the ASMR, ASPR, and ASDR of PAH into consecutive 5-year age intervals, spanning from 0–4 to 95–99 years. Similarly, the period from 1990 to 2021 was divided into 5-year intervals using the APC Web Tool, in accordance with the APC framework’s requirement for uniform age and period intervals (24). For forecasting, we employed the Bayesian Age-Period-Cohort (BAPC) model, which has demonstrated greater accuracy in comparison to several other linear power models, thus providing more reasonable predictive outcomes.

## Results

### Global and China trends in incidence, mortality and DALYs of PAH

As per GBD 2021 study, there was a significant rise in the incidence of PAH in China, climbing from 5,126 cases (95% CI: 4,139-6,211) in 1990 to 9,257 cases (95% CI: 7,350-11,508) in 2021, marking an 80.59% growth. On a global scale, the incidence saw an 85.62% increase, jumping from 23,301 cases (95% CI: 19,037-27,809) in 1990 to 43,251 cases (95% CI: 34,705-52,441) in 2021, even though the ASIR showed no notable variation between these years. Concurrently, the prevalence of PAH in China escalated by 86.74%, reaching 41,135 cases (95% CI: 32,839-51,357) by 2021, and globally, it grew by 81.46% to 191,808 cases (95% CI: 155,357-235,787). The ASPR in China experienced a minor uptick, from 2.07 per 100,000 (95% UI 1.68–2.54) in 1990 to 2.24 per 100,000 (95% UI 1.81–2.75) in 2021.

The mortality due to PAH in 2021 stood at 7,318 deaths (95% CI: 4,836-9,076), an 80.29% surge since 1990. Globally, the mortality rate rose by 48.37% over the same period. In terms of DALYs, the ASDR dropped globally from 13.21 (95% CI: 10.78–15.36) per 100,000 individuals in 1990 to 8.95 (95% CI: 6.04–11.13) per 100,000 individuals in 2021. Similarly, in China, the ASDR fell from 16.18 (95% CI: 12.63–21.6) per 100,000 individuals in 1990 to 8.24 (95% CI: 7.14–9.39) per 100,000 individuals in 2021(Table 1).

**Table 1:** Analysis and comparison of the trends in burden of PAH in China and worldwide from 1990 to 2021.

Location	Measure	1990		2021	
		All-ages cases	Age-standardized rates per 100,000 people	All-ages cases	Age-standardized rates per 100,000 people
		*n* (95% CI)	*n* (95% CI)	*n* (95% CI)	*n* (95% CI)
China	Incidence	5,126(4,139-6,211)	0.51 (0.41–0.62)	9,257 (7,350-11,508)	0.50 (0.40–0.60)
	Prevalence	22,028 (17,840-27,321)	2.07 (1.81–2.75)	41,135 (32,839-51,357)	2.24 (1.68–2.54)
	Deaths	4,059 (3,099-5,452)	0.61 (0.46–0.83)	7,318 (4,836-9,076)	0.42 (0.28–0.51)
	DALYs	149,699 (115,904-202,546)	16.18 (12.63–21.6)	150,941 (99,583-186,503)	8.24 (7.14–9.39)
Global	Incidence	23,301(19,037-27,809)	0.50 (0.40–0.60)	43,251 (34,705-52,441)	0.52 (0.42–0.62)
	Prevalence	105,703 (86,381-130,334)	2.30 (1.87–2.82)	191,808 (155,357-235,787)	2.28 (1.85–2.80)
	Deaths	14,842 (12,370-17,485)	0.35 (0.29–0.42)	22,021 (18,239-25,352)	0.27 (0.23–0.32)
	DALYs	687,419 (535,241-813,086)	13.21 (10.78–15.36)	642,104 (552,273-728,993)	8.95 (6.04–11.13)

DALYs, disability-adjusted life years.

The ASIR and ASPR change with an AAPC of −0.07 and 0.25%, respectively. Conversely, the ASDR and ASMR exhibited a downward trend since 1990, with an AAPC of −1.90% and − 1.26%, respectively. The AAPC of ASPR, ASIR, ASDR and ASMR of the global was 0.10, −0.03%, −0.82%, and - 1.52%, respectively (Table 1).

### Joinpoint regression analysis of the burden of PAH in China and worldwide

**Figure 1:**
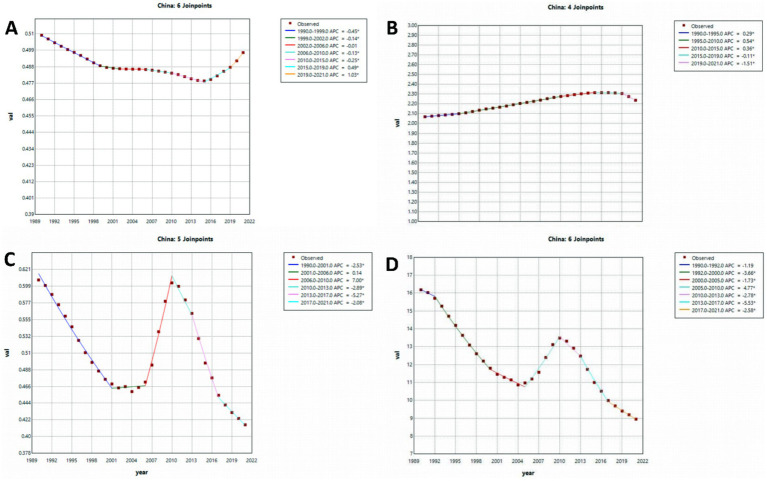
The APC of ASIR, ASPR, ASMR, and ASDR of PAH in China from 1990 to 2019 (* means *p*-values < 0.05 and significant results). **(A)** ASIR; **(B)** ASPR; **(C)** ASMR; **(D)** ASDR. Abbreviations: APC, age–period–cohort, ASIR age-standardized incidence rate, ASPR age-standardized prevalence rate, ASMR age-standardized mortality rate, ASDR age-standardized DALY rate, DALYs disability-adjusted life years.

The Joinpoint regression analysis, as illustrated in Figure 1 and Supplementary Figure S1, presents ASPR, ASIR, ASDR, and ASMR for PAH in China and globally from 1990 to 2021. In China, the APC for PAH ASIR indicated minor decreases between 1990 and 2015 (ASIR: 1990–1999 APC = −0.45; 1999–2002 APC = −0.14, *p* < 0.05; ASPR: 2006–2010 APC = −0.13; 2010–2015 APC = −0.25, *p* < 0.05). Conversely, an ascending trend with oscillations was observed from 2016 to 2021. Internationally, the ASIR for PAH demonstrated a significant rise between 1990–2000 and 2015–2021 (*p* < 0.05). Throughout 1990 to 2021, both Chinese and global ASMR for PAH exhibited notable reductions (*p* < 0.05). Regarding ASPR, a stable trend was observed from 1990 to 2021. Collectively, from 1990 to 2021, ASMRs and ASDRs in China and globally displayed significant decreases, albeit with intermittent fluctuations for both ASMRs between 2007 and 2013.

### Trends in the burden of PAH in China and worldwide

The ASDR for PAH in China and globally has seen a gradual decline from 1990 to 2021, with a more pronounced decrease in China. Additionally, China’s ASPR of PAH exhibited a modest upward trend during the same timeframe. In contrast, the ASIR and ASPR for PAH have remained relatively stable in both China and worldwide over the years (Supplementary Figure S2).

### Burden of PAH in different age groups in China in 1990 and 2021

**Figure 2:**
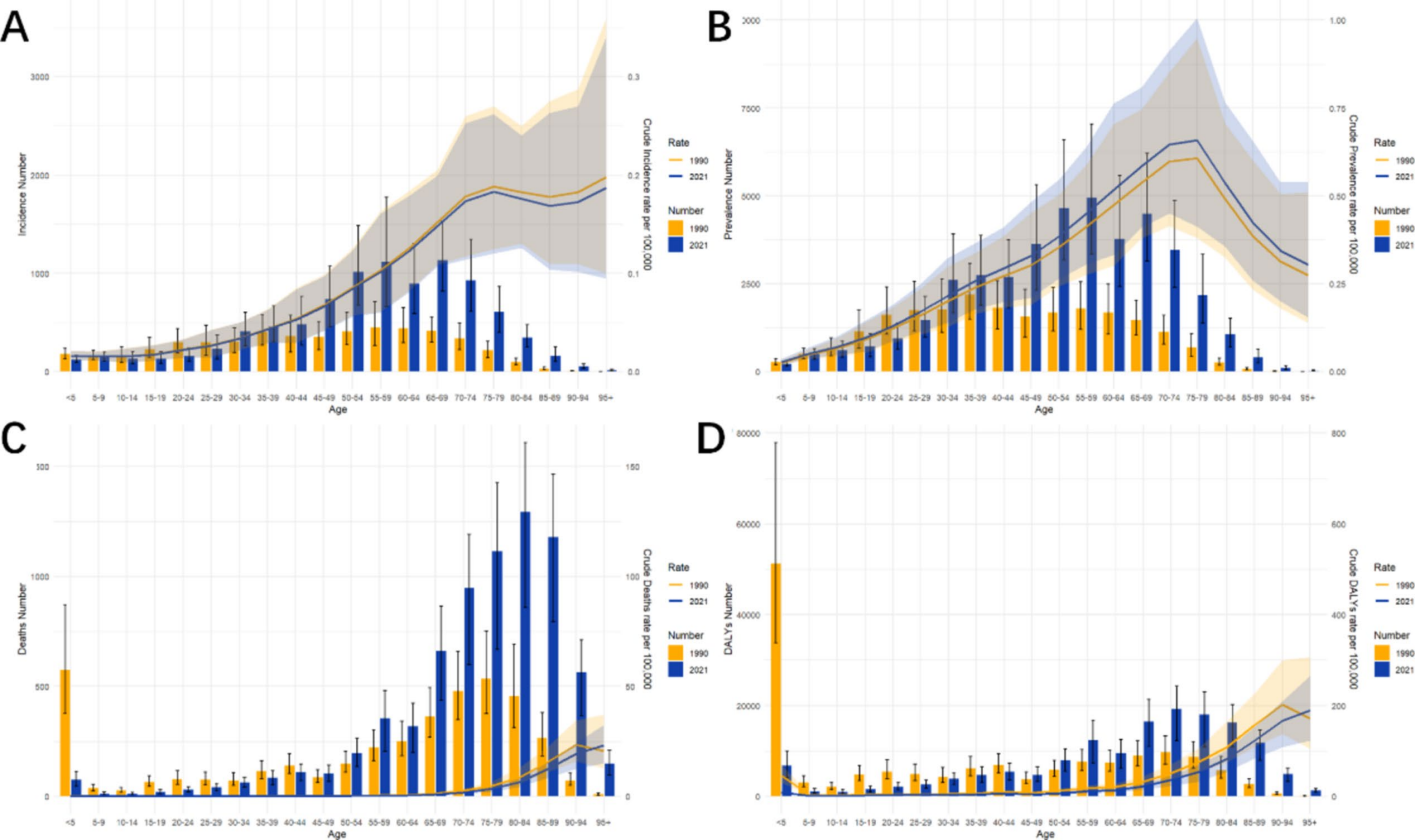
Comparative of the incidence, prevalence, deaths, and DALYs counts, along with their crude rates, by age group in China from 1990 and 2021. **(A)** Incident cases and CIR; **(B)** Prevalent cases and CPR; **(C)** Death cases and CMR; **(D)** DALYs counts and CDR; lines represent crude rates. Abbreviations: CIR crude incidence rate, CPR crude prevalence rate, CMR crude mortality rate, CDR crude DALYs rate, DALYs disability-adjusted life years.

Figure 2 presents a comparative analysis of PAH prevalence, incidence, mortality, and DALYs across various age demographics in China for the years 1990 and 2021, including a breakdown of crude rates. The incidence rate data revealed that PAH was more common among individuals over 45 years of age, peaking in the 50–70 age bracket. In both time points, the crude incidence and prevalence rates of PAH in China increased progressively from the 0–5 age group to the 70–74 age group, with the most cases noted in the 70–75 age range, albeit with a lower prevalence rate than that observed in China (Figures 2A,B and Supplementary Figure S3).

Mortality-wise, the 0–5 age group saw the highest number of deaths in 1990, shifting to the 80–84 age group in 2021. The crude mortality rates for PAH escalated with age, displaying an upward trajectory. The 95+ age group exhibited the highest mortality rate in both 1990 and 2019. Corresponding trends were identified in the crude DALY rates, which rose with increasing age. In 1990, the highest DALYs were found in the 0–5 age group, shifting to the 70–74 age group by 2021.

### Gender disparities in the burden of PAH in different age groups in China

**Figure 3:**
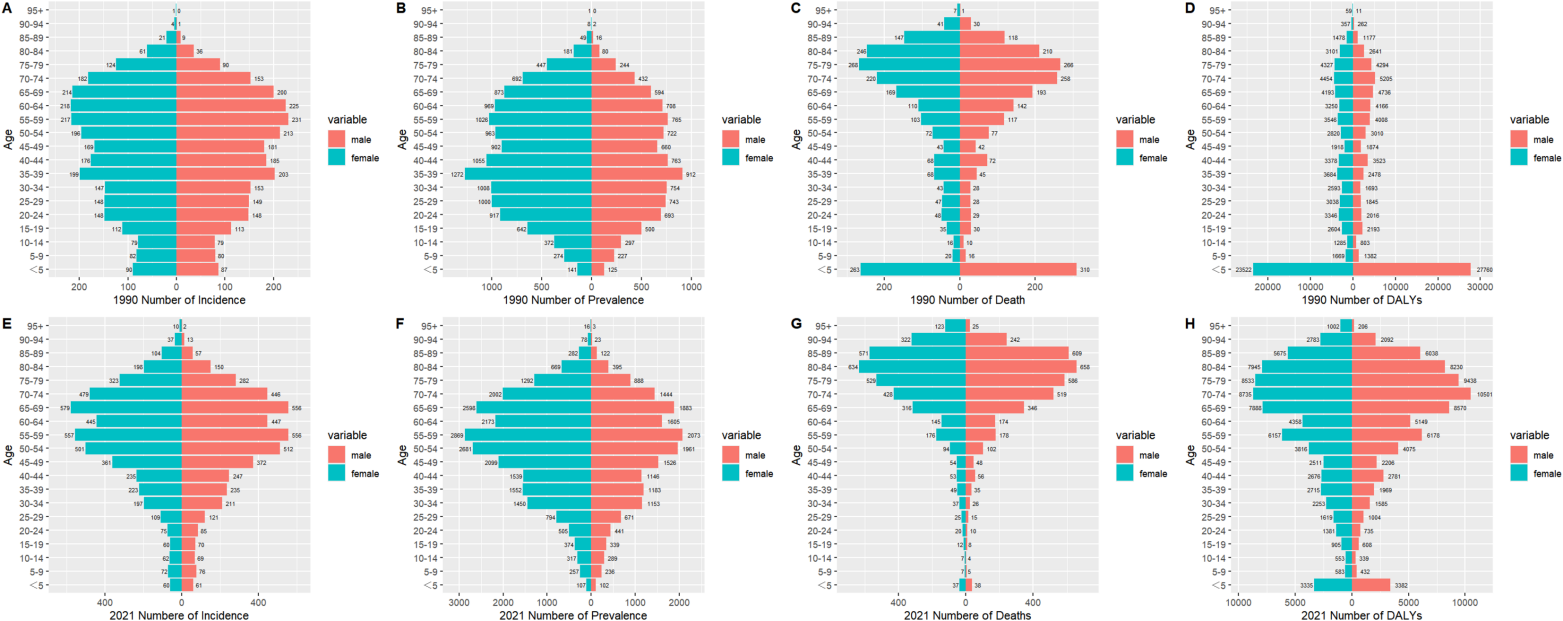
Comparison of the number of incidence, prevalence, mortality, and DALYs of PAH in males and females of different age groups in China in 1990 and 2021. **(A)** Incidence in 1990; **(B)** Prevalence in 1990; **(C)** Mortality in 1990; **(D)** DALYs in 1990; **(E)** Incidence in 2021; **(F)** Prevalence in 2021; **(G)** Mortality in 2021; **(H)** DALYs in 2021. Abbreviations: PAH Pulmonary arterial hypertension, DALYs disability-adjusted life years.

Figure 3 illustrates the assessment of the health impact and standardized disease metrics for PAH across different age groups and genders in China, spanning the years 1990 to 2021. The incidence data highlighted that the apex of PAH incidence for both genders was within the 50–70 age range. The prevalence findings from 1990 indicated a rise in PAH cases for both males and females, escalating from the 0–5 age group to the 35–39 age group, with the apex in prevalence rates noted in the 35–39 age group, which was similar to the global trend. In 1990, females exhibited a higher incidence of PAH across all age groups when compared to males in China and worldwide. In 2021, among all other age groups, females exhibited a higher incidence of PAH compared to males, with the highest rate observed in the 55–59 age group.

Mortality figures from 1990 revealed that males experienced a greater number of deaths than females across most age groups in China, with the highest mortality rates for both genders occurring at 0–5 age group. In 2021, males again showed a higher death toll than females, particularly in the elderly population above 50, with the maximum mortality rate observed in the 80–84 age group. The results for DALYs mirrored the mortality trends, showing that females had a higher DALY count than males across all age groups, with the exception of the 0–5 age group. The peak of DALYs for both genders in 1990 was noted in the 0–5 age group, shifting to the 70–74 age group by 2021 (Figure 3 and Supplementary Figure S4).

Supplementary Figure S5 presents an analysis of the disease burden and age-standardized metrics of PAH among males and females of all ages in China from 1990 to 2021. As depicted in Supplementary Figure S5A, ASIR of PAH for both genders peaked in 1990, followed by minor fluctuations. The ASPR for PAH in females and males rose from 1990 to 2018, with a slight decline observed from 2018 to 2021. Females consistently exhibited a higher ASPR compared to males, aligning with the global trend (Supplementary Figure S5). Furthermore, Supplementary Figure S6 illustrates that in 2010, the mortality rates, including the ASMR and ASDR, varied significantly between males and females, with males recording a notably higher ASMR. This gender disparity narrowed over time, coinciding with an overall decline in ASMR and ASDR. Conversely, the global ASIR and ASPR for PAH in both genders showed a gradual decrease from 1990 to 2021, which contrasts with the pattern observed in China.

### Descriptive analysis of PAH prevalence and mortality by age, period, and birth cohort groups

**Figure 4:**
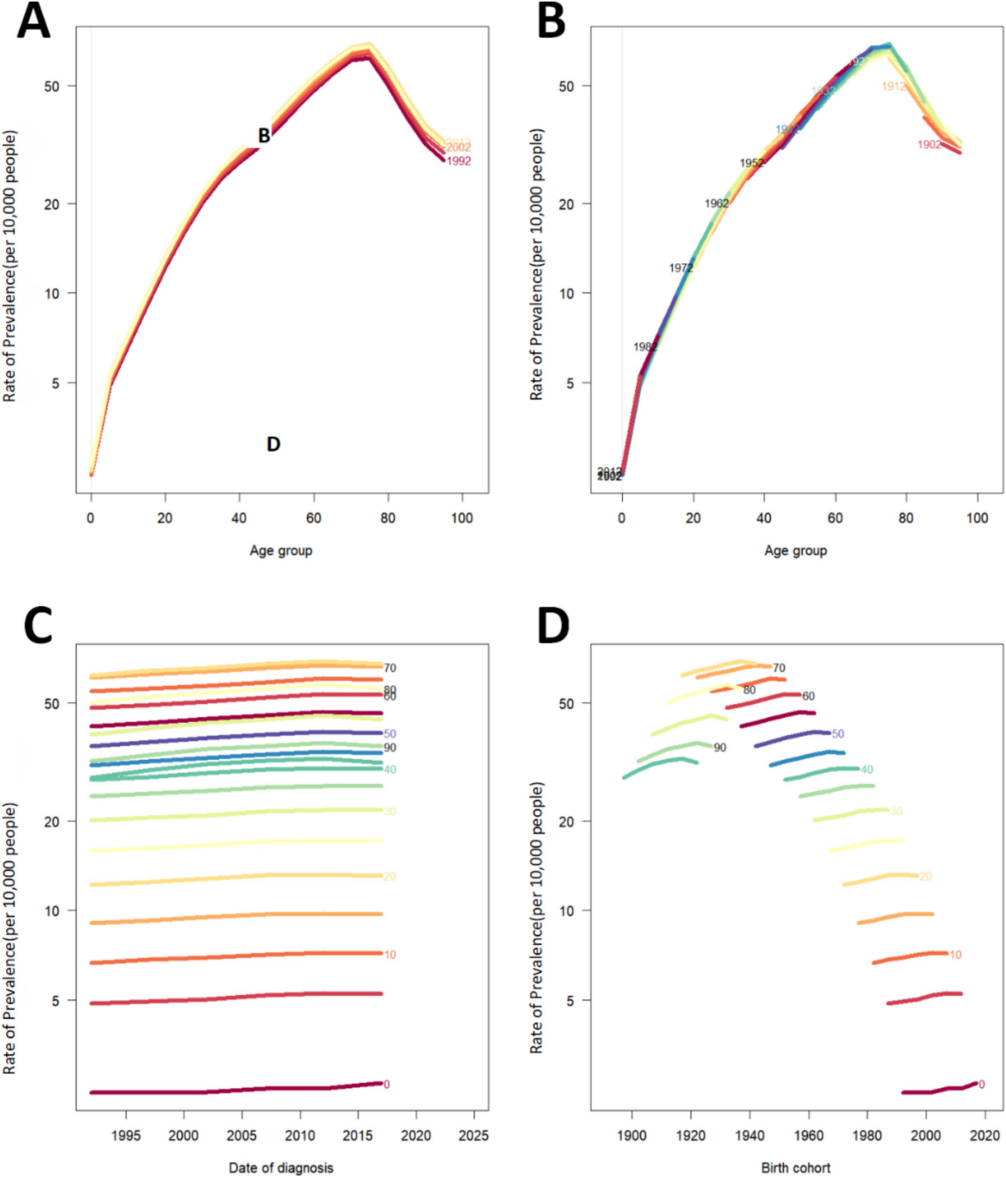
Prevalence rates of PAH in China. **(A)** The age-specific prevalence rates of PAH according to time periods; each line connects the age-specific prevalence for a 5-year period. **(B)** The age-specific prevalences rates of PAH according to birth cohort; each line connects the age-specific prevalence for a 5-year cohort. **(C)** The period-specific prevalence rates of PAH according to age groups; each line connects the birth cohort-specific prevalence for a 5-year age group. **(D)** The birth cohort-specific prevalence rates of PAH according to age groups; each line connects the birth cohort-specific prevalence for a 5-year age group. PAH Pulmonary arterial hypertension.

Figure 4 depicts the shifts in the prevalence and mortality rates of PAH in China and globally, categorized by age, time period, and birth cohort. The prevalence escalated with advancing age, reaching a zenith within the 70 to 75 years age bracket, followed by a slight decline post-75 years. Among individuals aged over 70, a notable rise in ASPR was observed from 1995 to 2010, contrasting with minor fluctuations in younger age groups. The latest birth cohort exhibited the lowest incidence of PAH. With increasing age, ASMR showed a significant decreased during 0–10 age group, and after the period, the rates gradually increased. Over time, there has been a rise in PAH prevalence within the older demographic. A downward trajectory in PAH-related mortality is observed across different birth cohorts, suggesting a reduced likelihood of PAH mortality for the most current birth cohort, with the exception of the 0–5 years age group. (Figure 4 and Supplementary Figure S7).

### Projections of the global trends of PAH until 2036

**Figure 5:**
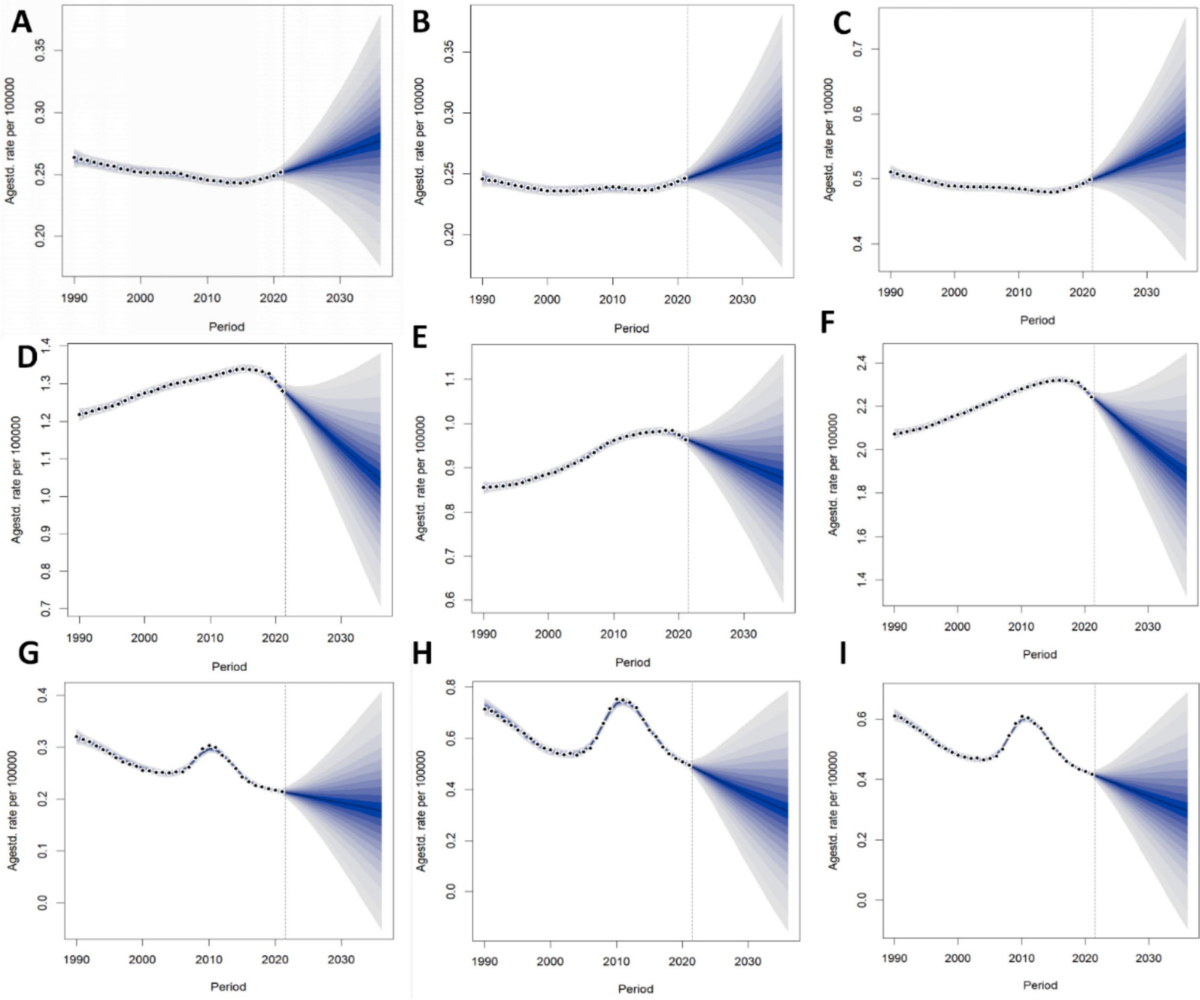
Projections of ASIR [**(A)**: male, **(B)**: female, **(C):** both], ASPR [**(D)**: male, **(E)**: female, **(F)**: both], and ASMR [**(G)**: male, **(H)**: female, **(I)**: both] of PAH among both sexes, men and women, until 2036. Abbreviations: PAH Pulmonary arterial hypertension, ASIR age-standardized incidence rate, ASPR age-standardized prevalence rate, ASMR age-standardized mortality rate.

Figure 5 forecasts that the ASPR, ASMR and ASIR for PAH are expected to remain relatively stable with minimal fluctuations in the forthcoming decade. It anticipates that by 2036, the rates for both incidence and mortality will be comparable between males and females. Regarding ASPR, it is projected to be higher in women compared to men in 2036. The ASIR is expected to experience a slight rise, from 0.50 per 100,000 in 2022 to 0.56 per 100,000 by 2036; the ASPR is predicted to show a gentle decline, from 2.22 per 100,000 (95% CI 2.13 to 2.21) in 2022 to 1.89 per 100,000 (95% CI 1.61 to 2.55) in 2036; and the ASMR is also foreseen to diminish slightly, from 0.41 per 100,000 (95% CI 45.93 to 48.05) in 2022 to 0.30 per 100,000 (95% CI 36.97 to 57.66) in 2036.

## Discussion

This research investigates the changing patterns of PAH in China across three decades, marking the inaugural application of joinpoint analysis alongside the APC model to assess PAH epidemiology in the country. The ASIR, ASMR, and ASPR for PAH have either experienced a slight uptick or remained stable both in China and globally. Despite this, the overall counts of PAH cases and fatalities have risen, likely attributable to the expanding global population and the phenomenon of aging, as reflected in the age-standardized data. Within China, the prevalence, incidence, mortality and DALYs associated with PAH exhibit correlations with the patients’ age and gender. PAH tends to be more common in older individuals, with higher incidence rates in the elderly and increased mortality rates among children aged 0–5. Regarding gender differences, females appear to be more prone to developing PAH but exhibit a lower mortality risk post-infection compared with their male counterparts.

During the last several decades, the enhancement of global living conditions and healthcare availability have led to a reduction in the disease burden of PAH in China (25). Advances in medical technology have augmented the capacity of healthcare providers to treat PAH more effectively (26). Li et.al. (27) report a marked enhancement in survival rates for individuals suffering from PAH in China, as contrasted with the data from the 2007 registry. In our analysis, the 0–5 age group experienced the highest mortality figures in 1990. PAH, a severe pulmonary vascular disease, is known for its significant morbidity and mortality rates among pediatric populations. The causes of PAH in children are markedly different from those in adults, with the etiologies primarily categorized between idiopathic pulmonary arterial hypertension (IPAH) and developmental lung conditions (28, 29). However, when contrasted with global DALY figures, China exhibits relatively lower absolute numbers (30). Factors such as rapid economic development and rising educational levels are probable contributors to these observed improvements (30).

The stratified age analysis indicates that the incidence peak of PAH in China is currently centered around the 70–75 years age bracket. The prevalence of PAH, which peaks in the 70–80 years age group, aligns with global patterns. Data from a Chinese PAH registry spanning 1999 to 2004, showing a mean patient age of 35.9 ± 12.2 years (31), suggests a rising average age of PAH patients, signifying a crucial consideration in the treatment and management strategies for PAH (30). In the context of an aging population, the societal impact of chronic conditions like PAH merits serious attention.

In China, the prevalence associated with PAH are higher among women compared to men, corroborating previous research (32, 33). The NIH cohort reported that women have a 1.8-fold higher likelihood of developing PAH compared to men (34). However, women with PAH generally exhibit a more favorable prognosis than their male counterparts (35), potentially due to the beneficial role of estrogen. Concurrently, existing guidelines explicitly advise against pregnancy in women with PAH and recommend termination if pregnancy occurs (36–38).

Our forecast indicated that the ASPR, ASMR, and ASIR of PAH are likely to remain relatively stable with minor fluctuations in the forthcoming decade. Considering the universal trend of population aging, the growing proportion of elderly individuals could lead to a rise in PAH incidence. As previously highlighted, PAH continues to pose a significant global health challenge, and reducing its disease burden is of paramount importance. The ongoing pursuit of research into innovative therapies for PAH is essential for further enhancing patient outcomes (39).

This study had some limitations. The diagnosis of PAH is complicated because of its rarity and occult nature. Due to logistical and cost constraints of right heart catheterization (RHC), echocardiography remains the most widely used screening and diagnostic tool worldwide, even though RHC is a reliable diagnostic tool for PAH (40). However, echocardiography may be inaccurate as it may underestimate or overestimate the actual mean pulmonary artery pressure (41). Therefore, the misdiagnosis rate of PAH is high, and further research on the identification of PAHs risk factors is needed to help the early screening and prevention of PAH. It is also essential to consider the interaction between these risk factors and the potential for multiple factors to contribute to disease development in individual patients. Additionally, the absence of a comprehensive national PAH registry in China points to a persistent requirement, and estimates derived from sophisticated statistical models may carry inaccuracies (42). There is a necessity for large-scale cohort studies in China to accurately assess the local disease burden of PAH (43). To mitigate the escalating burden of PAH, Chinese healthcare systems should concentrate on innovative preventative strategies for PAH.

## Data Availability

The datasets presented in this study can be found in online repositories. The names of the repository/repositories and accession number(s) can be found in the article/supplementary material.

## Supplementary material

SUPPLEMENTARY FIGURE S1 The APC of ASIR, ASPR, ASMR, and ASDR of PAH in China from 1990 to 2019 (* means p-values < 0.05 and significant results). **(A)** ASIR; **(B)** ASPR; **(C)** ASMR; **(D)** ASDR. APC age–period–cohort, ASIR age-standardized incidence rate, ASPR age-standardized prevalence rate, ASMR age-standardized mortality rate, ASDR age-standardized DALY rate, DALYs disability-adjusted life years.

SUPPLEMENTARY FIGURE S2 Trend comparison of ASIR, ASPR, ASMR, and ASDR of PAH in China **(A)** and worldwide **(B)** from 1990 to 2021. PAH Pulmonary arterial hypertension, ASIR, age-standardized incidence rate; ASMR: age-standardized mortality rate; ASPR: age-standardized prevalence rate; DALYs Rate: disability-adjusted life years rate.

SUPPLEMENTARY FIGURE S3 Comparative of the incidence, prevalence, deaths, and DALYs counts, along with their crude rates, by age group globally from 1990 and 2021. **(A)** Incident cases and CIR; **(B)** Prevalent cases and CPR; **(C)** Death cases and CMR; **(D)** DALYs counts and CDR. CIR crude incidence rate, CPR crude prevalence rate, CMR crude mortality rate, CDR crude DALYs rate, DALYs disability-adjusted life years.

SUPPLEMENTARY FIGURE S4 Comparison of the number of incidence, prevalence, mortality, and DALYs of PAH in males and females of different age groups globally in 1990 and 2021. **(A)** Incidence in 1990; **(B)** Prevalence in 1990; **(C)** Mortality in 1990; **(D)** DALYs in 1990; **(E)** Incidence in 2021; **(F)** Prevalence in 2021; **(G)** Mortality in 2021; **(H)** DALYs in 2021. PAH Pulmonary arterial hypertension, DALYs disability-adjusted life years.

SUPPLEMENTARY FIGURE S5 Comparison of full-age cases and age-standardized rates of incidence, prevalence, mortality and DALYs among men and women in China from 1990 to 2021. **(A)** Incident cases and ASIR; **(B)** Prevalent cases and ASPR; **(C)** Death cases and ASMR; **(D)** DALYs counts ASDR. Bar charts represent counts; lines represent age-standardized rates. ASIR age-standardized incidence rate, ASPR age-standardized prevalence rate, ASMR age-standardized mortality rate, ASDR age-standardized DALY rate, DALYs disability-adjusted life years.

SUPPLEMENTARY FIGURE S6 Comparison of full-age cases and age-standardized rates of incidence, prevalence, mortality and DALYs among men and women worldwide from 1990 to 2021. **(A)** Incident cases and ASIR; **(B)** Prevalent cases and ASPR; **(C)** Death cases and ASMR; **(D)** DALYs counts ASDR. Bar charts represent counts; lines represent age-standardized rates. APC age–period–cohort, ASIR age-standardized incidence rate, ASPR age-standardized prevalence rate, ASMR age-standardized mortality rate, ASDR age-standardized DALY rate, DALYs disability-adjusted life years.

SUPPLEMENTARY FIGURE S7 Mortality rates of PAH in China. **(A)** The age-specific mortality rates of PAH according to time periods; each line connects the age-specific mortality for a 5-year period. **(B)** The age-specific mortality rates of PAH according to birth cohorts; each line connects the age-specific mortality for a 5-year cohort. **(C)** The period-specific mortality rates of PAH according to age group; each line connects the birth cohort-specific mortality for a 5-year age group. **(D)** The birth cohort-specific mortality rates of PAH according to age groups; each line connects the birth cohort-specific mortality for a 5-year age group. PAH Pulmonary arterial hypertension.
