# Supplementary figures and images for: Long-term trends in the burden of pulmonary arterial hypertension in China and worldwide: new insights based on GBD 2021
Source: Front Med (Lausanne). 2025 Jan 7;11:1502916. doi: 10.3389/fmed.2024.1502916 (PMC11748298; doi:10.3389/fmed.2024.1502916)

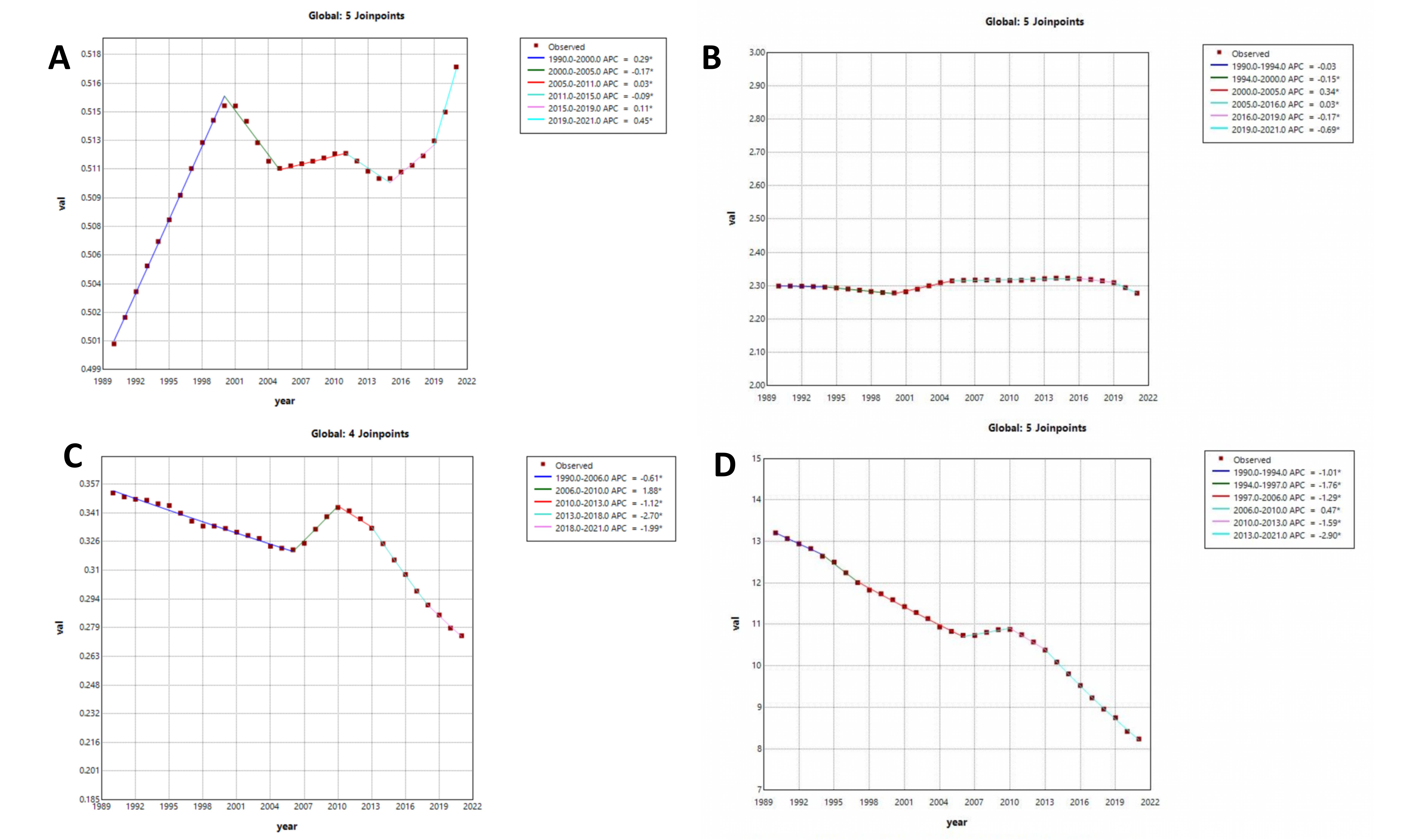

Supplement: SUPPLEMENTARY FIGURE S1 — The APC of ASIR, ASPR, ASMR, and ASDR of PAH in China from 1990 to 2019 (* means p-values < 0.05 and significant results). (A) ASIR; (B) ASPR; (C) ASMR; (D) ASDR. APC age–period–cohort, ASIR age-standardized incidence rate, ASPR age-standardized prevalence rate, ASMR age-standardized mortality rate, ASDR age-standardized DALY rate, DALYs disability-adjusted life years. [file Image_1.JPEG]

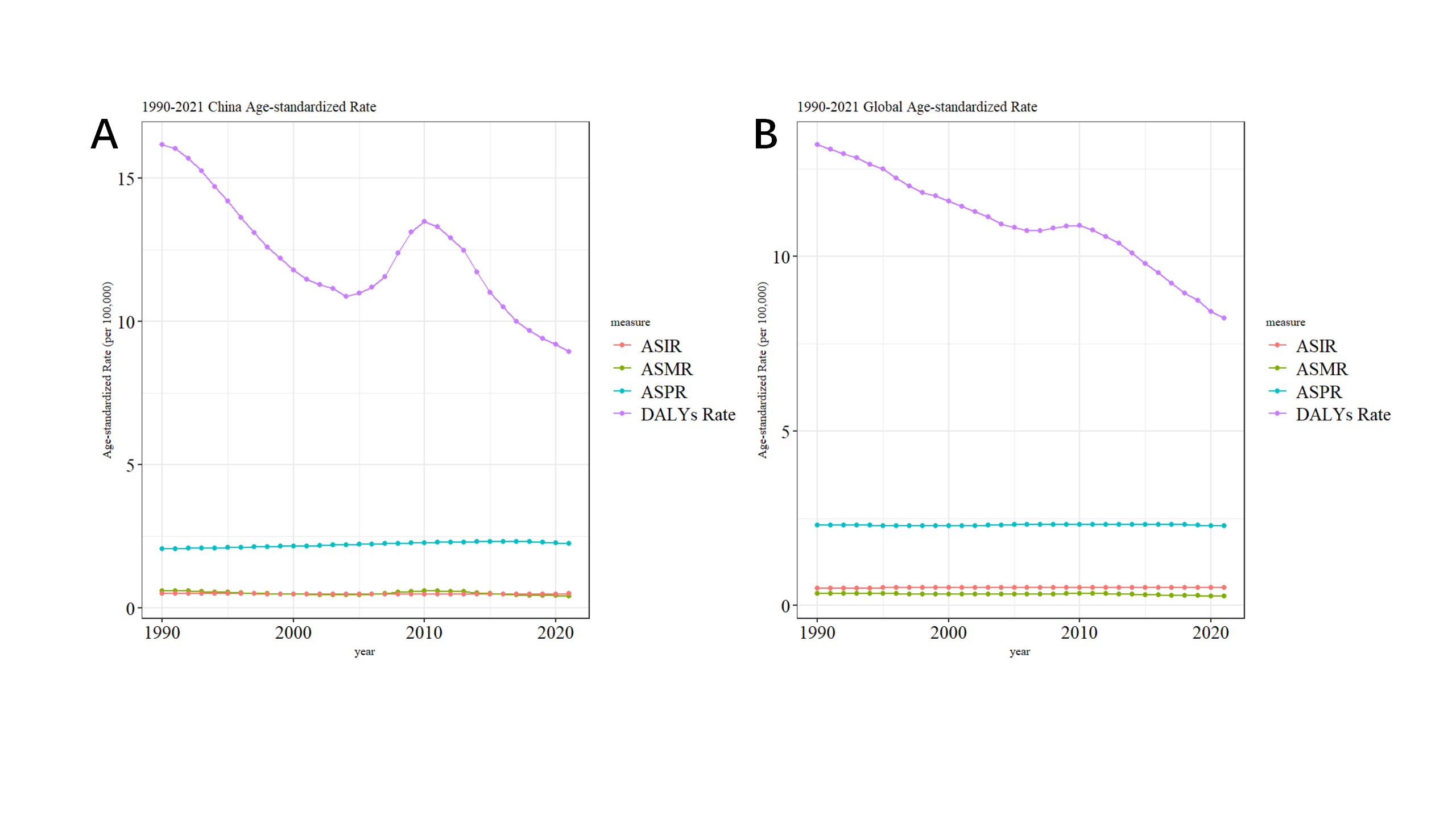

Supplement: SUPPLEMENTARY FIGURE S2 — Trend comparison of ASIR, ASPR, ASMR, and ASDR of PAH in China (A) and worldwide (B) from 1990 to 2021. PAH Pulmonary arterial hypertension, ASIR, age-standardized incidence rate; ASMR: age-standardized mortality rate; ASPR: age-standardized prevalence rate; DALYs Rate: disability-adjusted life years rate. [file Image_2.TIF]

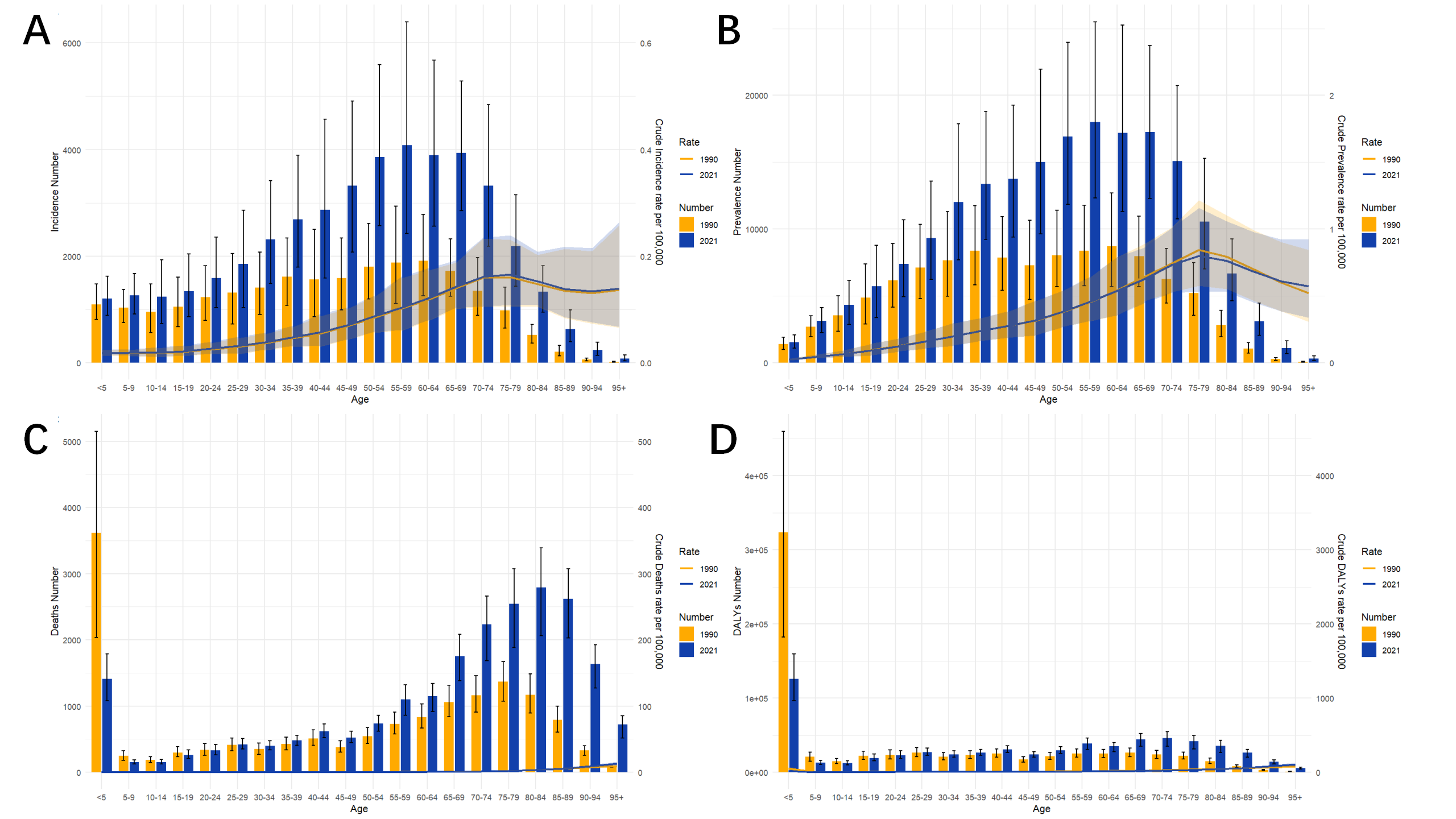

Supplement: SUPPLEMENTARY FIGURE S3 — Comparative of the incidence, prevalence, deaths, and DALYs counts, along with their crude rates, by age group globally from 1990 and 2021. (A) Incident cases and CIR; (B) Prevalent cases and CPR; (C) Death cases and CMR; (D) DALYs counts and CDR. CIR crude incidence rate, CPR crude prevalence rate, CMR crude mortality rate, CDR crude DALYs rate, DALYs disability-adjusted life years. [file Image_3.TIF]

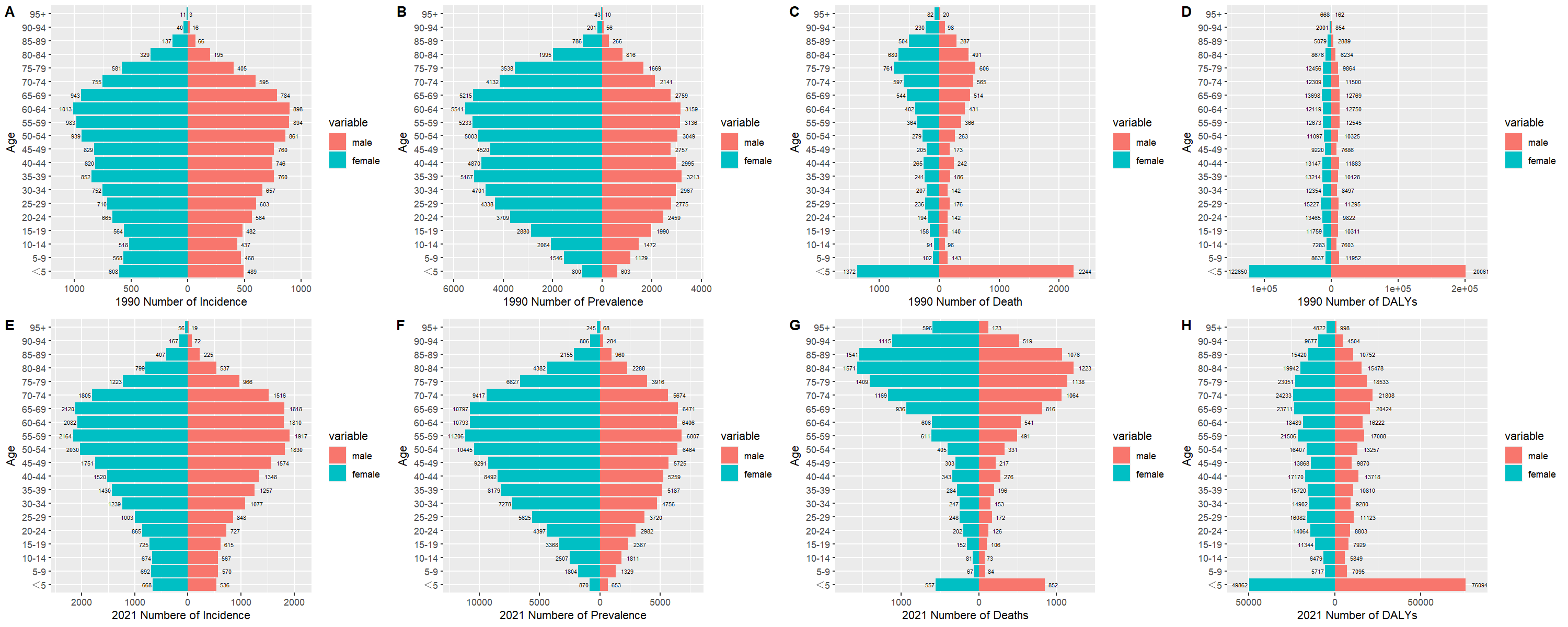

Supplement: SUPPLEMENTARY FIGURE S4 — Comparison of the number of incidence, prevalence, mortality, and DALYs of PAH in males and females of different age groups globally in 1990 and 2021. (A) Incidence in 1990; (B) Prevalence in 1990; (C) Mortality in 1990; (D) DALYs in 1990; (E) Incidence in 2021; (F) Prevalence in 2021; (G) Mortality in 2021; (H) DALYs in 2021. PAH Pulmonary arterial hypertension, DALYs disability-adjusted life years. [file Image_4.PNG]

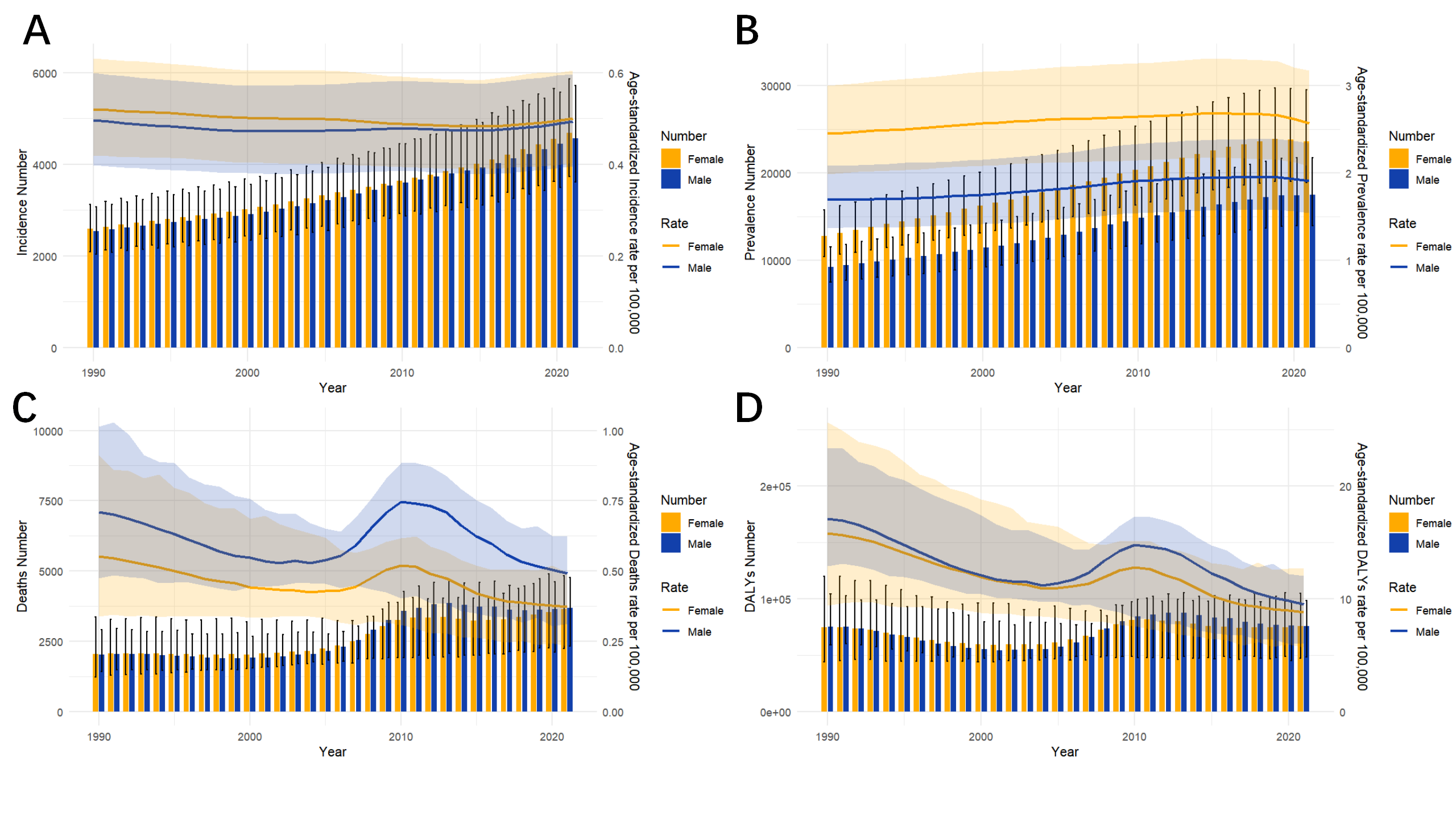

Supplement: SUPPLEMENTARY FIGURE S5 — Comparison of full-age cases and age-standardized rates of incidence, prevalence, mortality and DALYs among men and women in China from 1990 to 2021. (A) Incident cases and ASIR; (B) Prevalent cases and ASPR; (C) Death cases and ASMR; (D) DALYs counts ASDR. Bar charts represent counts; lines represent age-standardized rates. ASIR age-standardized incidence rate, ASPR age-standardized prevalence rate, ASMR age-standardized mortality rate, ASDR age-standardized DALY rate, DALYs disability-adjusted life years. [file Image_5.TIF]

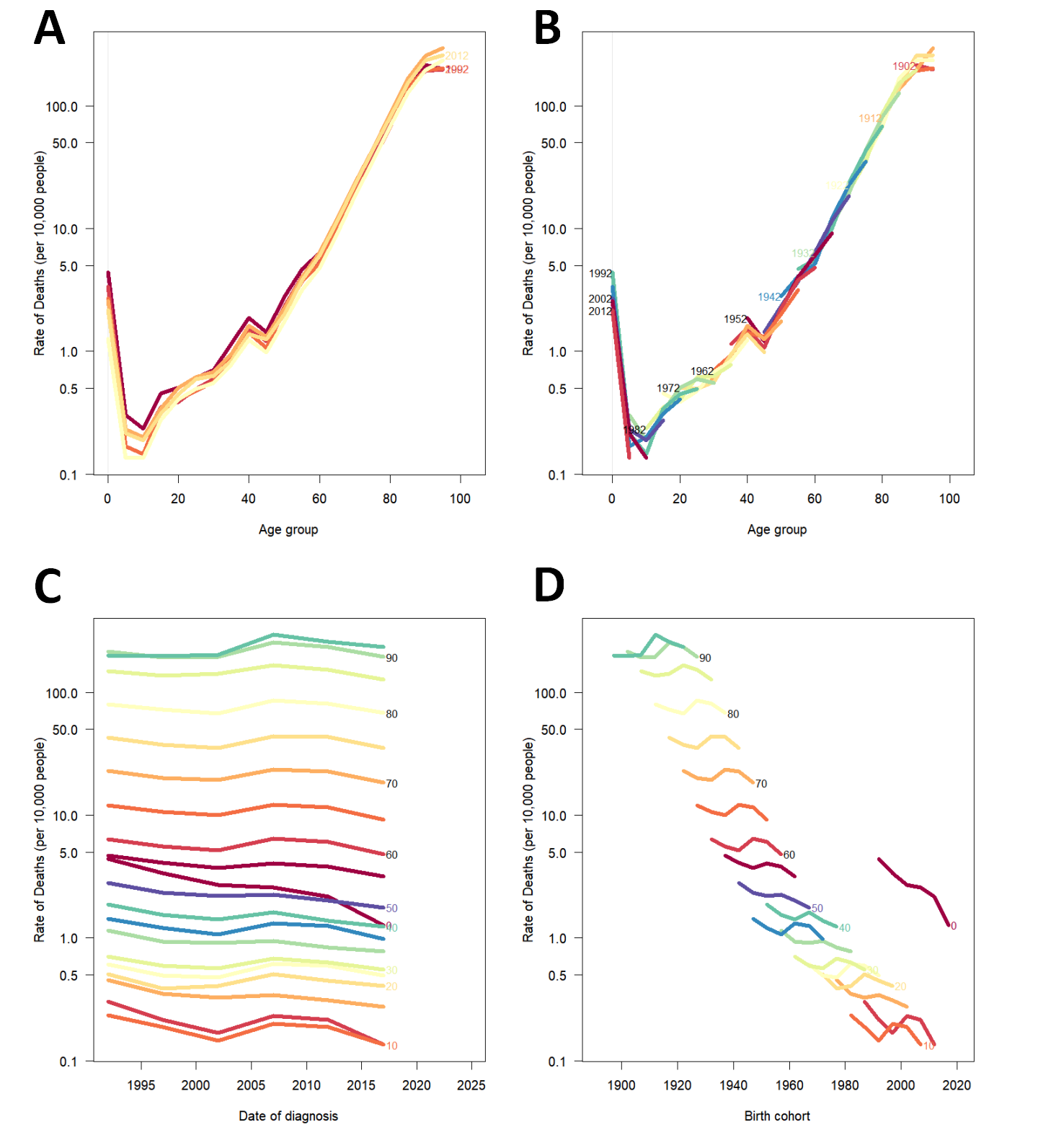

Supplement: SUPPLEMENTARY FIGURE S7 — Mortality rates of PAH in China. (A) The age-specific mortality rates of PAH according to time periods; each line connects the age-specific mortality for a 5-year period. (B) The age-specific mortality rates of PAH according to birth cohorts; each line connects the age-specific mortality for a 5-year cohort. (C) The period-specific mortality rates of PAH according to age group; each line connects the birth cohort-specific mortality for a 5-year age group. (D) The birth cohort-specific mortality rates of PAH according to age groups; each line connects the birth cohort-specific mortality for a 5-year age group. PAH Pulmonary arterial hypertension. [file Image_7.PNG]
